# Childhood IQ and survival to 79: Follow-up of 94% of the Scottish Mental Survey 1947

**DOI:** 10.1016/j.intell.2017.05.002

**Published:** 2017-07

**Authors:** Iva Čukić, Caroline E. Brett, Catherine M. Calvin, G. David Batty, Ian J. Deary

**Affiliations:** a Department of Psychology, University of Edinburgh, UK; b Centre for Cognitive Ageing and Cognitive Epidemiology, University of Edinburgh, UK; c Department of Epidemiology and Public Health, University College London, UK; d Natural Sciences and Psychology, Liverpool John Moors University, UK

**Keywords:** Childhood intelligence, IQ, All-cause mortality, SMS1947, Sex differences

## Abstract

**Objective:**

To extend previous literature that suggests higher IQ in youth is associated with living longer. Previous studies have been unable to assess reliably whether the effect differs across sexes and ages of death, and whether the effect is graded across different levels of IQ.

**Methods:**

We test IQ-survival associations in 94% of the near-entire population born in Scotland in 1936 who took an IQ test at age 11 (n = 70,805) and were traced in a 68-year follow-up.

**Results:**

Higher IQ at age 11 years was associated with a lower risk of death (HR = 0.80, 95% CI = 0.79, 0.81). The decline in risk across categories of IQ scores was graded across the full range with the effect slightly stronger in women (HR = 0.79, 95% CI = 0.77, 0.80) than in men (HR = 0.82, 95% CI = 0.81, 0.84). Higher IQ had a significantly stronger association with death before and including age 65 (HR = 0.76, 95% CI = 0.74, 0.77) than in those participants who died at an older age (HR = 0.79, 95% CI = 0.78, 0.80).

**Conclusions:**

Higher childhood IQ is associated with lower risk of all-cause mortality in both men and women. This is the only near-entire population study to date that examines the association between childhood IQ and mortality across most of the human life course.

## Highlights

• 94% of the participants of the Scottish Mental Survey 1947 were traced.
• Higher childhood IQ was related to a lower risk of all-cause mortality by age 79.
• The effect was slightly stronger in women than in men.
• The decline in risk across categories of IQ scores was graded across the full range.
• This is the only study of IQ and mortality in an entire year-of-birth cohort.

## 1 Introduction

It is now well-documented that higher childhood intelligence, as ascertained from standard tests, is associated with living longer. This is the case for both all-cause mortality (Batty et al., 2008b, Batty et al., 2009b, Hart et al., 2005, Whalley and Deary, 2001), and mortality from specific causes, particularly cardiovascular disease, (Batty et al., 2008a, Deary et al., 2010, Hemmingsson et al., 2009), accidents (Batty, Gale, Tynelius, Deary, & Rasmussen, 2009) and suicide (Batty et al., 2010, Gunnell et al., 2005). The most recent meta-analysis reported that a one standard deviation advantage in early life cognitive ability test scores is related to a 24% reduction in the risk of death during a follow-up of up to 69 years (Calvin et al., 2011). However, a number of questions regarding the associations between IQ and mortality remain under-explored. Most notably, with many samples either comprising only men (Batty et al., 2008a, Batty et al., 2009b, Hemmingsson et al., 2009), or being insufficiently powered to compute sex-specific effect estimates (Calvin et al., 2011), little is known about the link between pre-adult IQ and mortality in women. In one of the few exceptions, Whalley and Deary (Whalley & Deary, 2001) reported a stronger protective association of higher IQ and lower mortality in women, but could not rule out that this difference was cohort-specific due to a larger number of higher intelligence men dying in active service in the second world war. One report from a post-war cohort study did not detect an association between IQ and mortality in women (Kuh, Richards, Hardy, Butterworth, & Wadsworth, 2004), and the other one detected the association between IQ and mortality only at the age of 60 and above (Lager, Bremberg, & Vågerö, 2009).

Another important issue concerns the nature of the association between IQ and mortality. Whereas some studies showed that the association is graded across the whole range of IQ (Batty et al., 2009b, Hemmingsson et al., 2009, Whalley and Deary, 2001), others reported that it is driven by the accumulation of risk factors at the lower end of the IQ distribution (Kuh et al., 2004). Furthermore, higher childhood IQ may be protective against early deaths, but may not be associated with deaths in older age, as was found in one study (Hart et al., 2005). However, these studies were underpowered to reliably assess the associations across the whole range of cognitive ability, ages of death, and to examine potential sex differences in the IQ-mortality association.

In the present study we were able to address all of the above limitations. The data for our study are a near-entire year of birth cohort from Scotland, with a long period of follow-up and a large number of deaths. We assessed the association between childhood IQ and survival to age 79, whether the relationship is graded, and also if there was any differential effect with respect to sex.

## 2 Method

### 2.1 Sample

On June 4, 1947 almost all individuals born in 1936 and attending Scottish schools sat an intelligence test as part of the Scottish Mental Survey 1947 (SMS1947). Testing was conducted by the Scottish Council for Research in Education with an aim to assess the intelligence levels of the entire generation of children born in 1936 and attending schools in Scotland in June 1947, as part of a project to follow cross-generational changes in mean intelligence levels (Deary et al., 2009a, Maxwell, 1969). Intelligence test scores, from a paper-and-pencil test administered by teachers, were obtained for 70,805 children (50.6% boys). This was about 94% of the estimated 1936-born population of Scotland (*n* = 75,286). The remaining 6% or so did not attend school on the day of testing.

To ascertain mortality information for the whole year of birth, tracing was done using the National Health Service Central Register (NHSCR) in Dumfries for participants traceable in Scotland and Northern Ireland, and Health and Social Care Information Centre (HSCIC) in Southport for those traceable in England and Wales. Tracing was conducted using participants' date of birth, surname, forename, sex, and name and location of school.

**Fig. 1:**
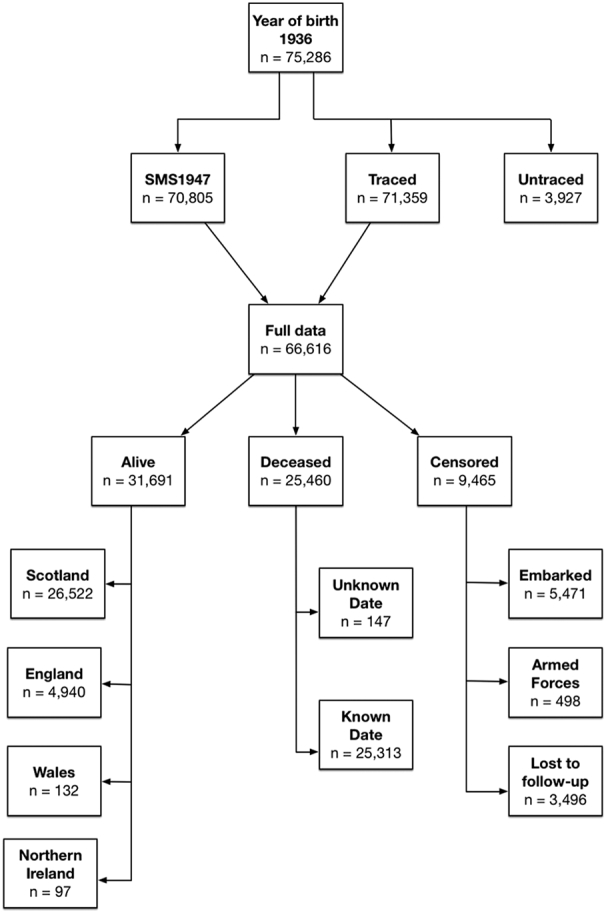
Sample composition and vital status at follow-up.

Vital status and death registration data were linked to participants with complete SMS1947 intelligence test scores. Intelligence test results and vital status data were available for 66,616 participants (51% men). That is, 94% of those who took part in the Scottish Mental Survey 1947 were traced and had childhood intelligence test data. A flow chart representing sample composition is presented in Fig. 1. Ethical approval for the study was obtained from Scotland A Research Ethics Committee (12-SS-0024). Support for linkage without consent was given under section 251 of the NHS Act 2006 by The Confidentiality Advisory Group of the Health Research Authority for participants traced in England and Wales (Ref. ECC 6-02(FT4 2012)), and by the Privacy Advisory Committee for participants traced in Scotland (Ref. 39–12) (Brett & Deary, 2014).

SMS1947 = Scottish Mental Survey 1947. Censored = unknown vital status at follow-up date. Reasons are given in squares below: Embarked = Emigrated abroad. Armed Forces = Joined Armed Forces. Lost to follow-up = No information available.

### 2.2 Measures

#### 2.2.1 Childhood intelligence

To assess childhood intelligence at age 11, the Moray House Test (MHT) no. 12. was used (Deary et al., 2012, Deary et al., 2009b, Deary et al., 2004). The test contains 71 items, including reasoning, word classification, analogies, and spatial orientation, and had a maximum possible score on the test of 76. The test was group-administered by teachers in classrooms and had a time constraint of 45 min. The MHT was concurrently validated in 1947 against the Terman-Merrill revision of the Binet scales (Deary et al., 2007). It has been well externally-validated since, and is a reliable measure of general intelligence (Deary et al., 2004, Deary et al., 2012) that shows high rank-order stability across the life-span (Deary, Whalley, Lemmon, Crawford, & Starr, 2000).

#### 2.2.2 Date of death

Vital status and date of death, where appropriate, were supplied by NHSCR Dumfries for participants traced in Scotland and Northern Ireland, and by HSCIC Southport for those located in England and Wales.

### 2.3 Analyses

The time-to-event variable (in days) was calculated using the participant's date of birth as a starting point. The censoring date is the date of the event that marks the end of the time-to-event variable, and it differs across participants as follows: for those known to be alive in England, Scotland, Wales and Northern Ireland censoring date was 26th June 2015 (end of follow-up). For those embarked (i.e. emigrated abroad), joined the Armed Forces and never re-registered with a general practitioner (GP), or otherwise lost to follow-up (i.e., cancelled their registration with a GP), the last known date of registration with a GP was used. For the deceased participants with known date of death, date of death is used as the end point of the time-to-event variable. For the deceased participants with an unknown date of death, the date of the last known GP registration was used as a censoring date. The exact number of participants for each of the categories is given in Fig. 1. Cox proportional hazard models were used to assess mortality risk associated with childhood IQ scores, controlling for age at the time of IQ testing. Hazard Ratios (HRs) were shown to illustrate change in risk both per 1-SD increase in IQ scores, and per IQ decile increase, and therefore are a relative measure of mortality risk. In all analyses, the lowest IQ category serves as a reference for the change in mortality risk. All analyses were computed in R environment, version 3.1.3 (R Core Team, 2015), using the ‘survival’ package (Therneau, 2015).

## 3 Results

Data from 66,616 people were included in the analysis. This is 88.5% of everyone born in Scotland in 1936, and 94.1% of those who took part in the SMS1947. Of these, 33,956 (51%) were men. A 68-year follow-up gave rise to 25,460 deaths (16,220 in men). Mean unadjusted childhood MHT scores for alive, deceased, and untraced participants are given in Table 1. In the whole sample, and in men and women separately, the deceased group had lower intelligence test scores than the group still alive, and the untraced group had the highest mean score.

**Table 1:** Mean age 11 Moray House Test score (SD) by participants' follow-up status up to 2015 for Scottish people born in 1936.

	Alive	Deceased	Censored	Untraced	*P*-Value for difference
All IQ	38.4 (15.4)	34.7 (16.0)	36.5 (14.9)	40.2 (16.3)	< 0.001
n	33,709	27,574	10,076	3927	
Men IQ	37.8 (16.2)	34.1 (16.4)	35.9 (15.5)	39.6 (17.1)	< 0.001
n	15,065	16,220	5050	1739	
Women IQ	38.8 (14.7)	35.6 (15.4)	37.2 (14.2)	40.4 (15.7)	< 0.001
n	18,644	11,354	5026	2188	

Higher childhood IQ (MHT) scores were associated with a lower risk of death by age 79 (HR = 0.80, 95% CI = 0.79, 0.81; Table 2). In the first model, we included age at IQ testing and IQ test score. In the next model, we controlled for the effects of sex. The association between childhood IQ and mortality remained significant, and similar in magnitude (HR = 0.81, 95% CI = 0.80, 0.82). As expected, women had lower mortality risk.

**Table 2:** Hazard Ratios (95% Confidence Intervals) for all-cause mortality risk by age 79 for Moray House Test (MHT) IQ scores at age 11 years for Scottish people born in 1936.

	Full sample		Men only		Women only	
	N = 66,616 (25,460 deaths)		N = 33,956 (15,007 deaths)		N = 32,660 (10,453 deaths)	
	HR (95% CI)	*p*	HR (95% CI)	*p*	HR (95% CI)	*p*
Model 1						
Age	1.03 (1.02, 1.05)	< 0.001	1.02 (1.01, 1.04)	< 0.01	1.04 (1.02, 1.06)	< 0.001
MHT IQ	0.80 (0.79, 0.81)	< 0.001	0.82 (0.81, 0.83)	< 0.001	0.79 (0.77, 0.80)	< 0.001
Model 2						
Age	1.03 (1.02, 1.04)	< 0.001	–		–	
Sex^a^	0.66 (0.64, 0.68)	< 0.001	–		–	
MHT IQ	0.81 (0.80, 0.82)	< 0.001	–		–	
Model 3						
Age	1.03 (1.02, 1.04)	< 0.001	–		–	
Sex^a^	0.65 (0.64, 0.67)	< 0.001	–		–	
MHT IQ	0.83 (0.81, 0.84)	< 0.001	–		–	
MHT IQ × sex^a^	0.95 (0.92, 0.97)	< 0.001	–		–	

Age = Age (days) at SMS1947. HRs for continuous variables are given per 1SD increase.

a Reference = male

In the next step, we focused on possible sex differences in the IQ-mortality associations. As shown in Table 2, the association of higher IQ (per 1-SD increase) with lower mortality was slightly stronger in women (HR = 0.79, 95% CI = 0.77, 0.80) than in men (HR = 0.82, 95% CI = 0.81, 0.84). This difference was statistically significant (*p*-value for interaction < 0.001) due to the high statistical power of our study, but relatively small in magnitude (6% per 1-SD increase in IQ).

**Fig. 2:**
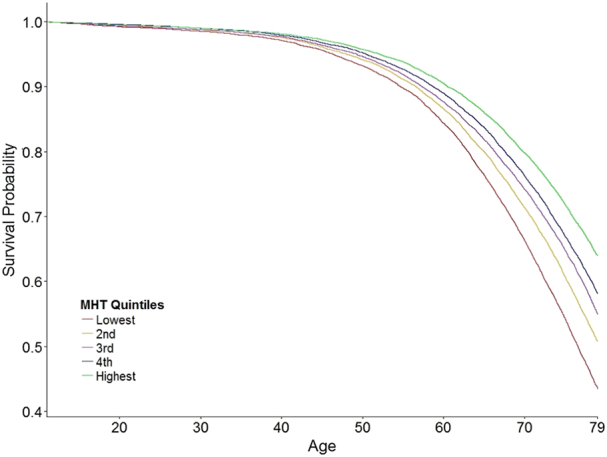
Survival curves for each of the Moray House Test IQ score quintiles for Scottish people born in 1936.

To assess whether the observed association between childhood IQ and mortality is graded over the whole range of IQ scores, we first split the sample into IQ quintiles. Survival curves for each of the quintiles are presented in Fig. 2, and suggest a graded decrease in risk as IQ category increases. To assess if this is true for both men and women, we categorised the MHT scores into deciles, and ran the same proportional hazard models as before, but with the categorised MHT scores as a predictor, in men and women separately. As shown in Table 3 and Fig. 2, there was a graded association between IQ and mortality in both men and women, and the effect was slightly stronger in women across the whole range of IQ. In men, the hazard ratio for the lowest decile was twice that for the highest, with an even stronger result in women.

**Table 3:** Hazard Ratios (95% Confidence Intervals) for all-cause mortality risk for each of the Moray House Test IQ score deciles for Scottish men and women born in 1936.

	Men			Women		
IQ Decile	N	Deaths	HR (95% CIs)	N	Deaths	HR (95% CIs)
1st (lowest)	4260	2356	1 (reference)	2743	1258	1 (reference)
2nd	3643	1916	0.93 (0.88, 0.99)	3203	1310	0.87 (0.81, 0.94)
3rd	3157	1556	0.86 (0.80, 0.91)	3102	1093	0.73 (0.67, 0.79)
4th	3319	1566	0.82 (0.77, 0.88)	3294	1137	0.72 (0.66, 0.78)
5th	3848	1671	0.74 (0.70, 0.79)	4044	1302	0.66 (0.61, 0.71)
6th	3275	1375	0.72 (0.68, 0.77)	3519	1054	0.60 (0.56, 0.66)
7th	3242	1323	0.69 (0.64, 0.74)	3542	1021	0.57 (0.53, 0.62)
8th	2979	1139	0.64 (0.59, 0.68)	3027	839	0.55 (0.50, 0.60)
9th	2878	1044	0.58 (0.54, 0.63)	3022	731	0.47 (0.43, 0.52)
10th	3355	1061	0.50 (0.47, 0.54)	3164	708	0.41 (0.38, 0.45)

*Note.* All models control for age in days at SMS1947.

*Note.* The MHT score was residualised on age at the time of testing.

We tested whether the association between IQ and mortality varied depending on age at death. We found that, adjusting for sex, the effect of IQ was slightly stronger in participants who died when they were 65 and younger (HR = 0.76, 95% CI = 0.74, 0.77) than in those participants who died when they were over 65 years old (HR = 0.79, 95% CI = 0.78, 0.80). The effect was similar for men and women who died before and including age 65 (HR = 0.78 and HR = 0.75, respectively), as well as for men and women who died when they were older than 65 (HR = 0.81 and 0.78, respectively). Although they were small in magnitude, they were all significantly different (all *p*s < 0.01). We based the initial cutoff score of 65 on a previously published study for comparison (Hart et al., 2005).

**Fig. 3:**
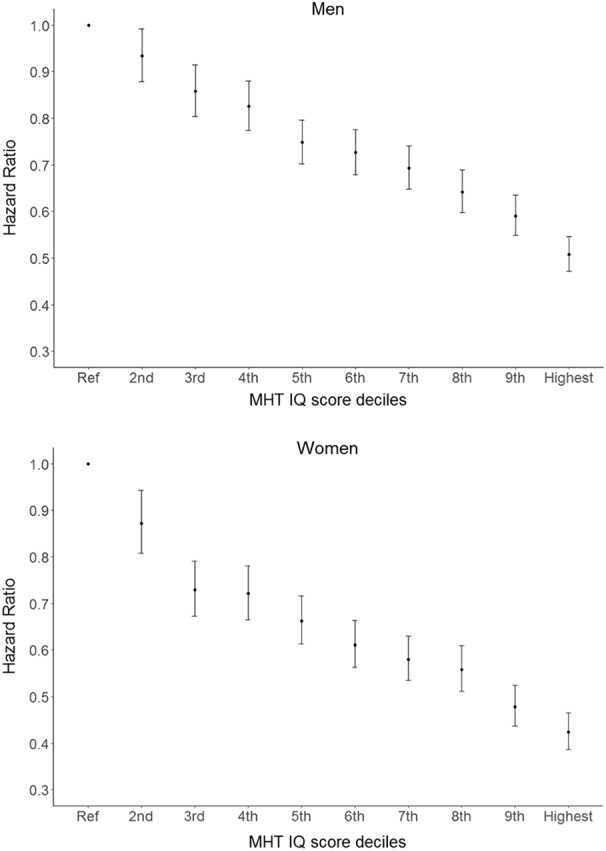
Survival hazard ratios with 95% Confidence Intervals for Scottish men and women for each of the Moray House Test IQ score deciles. Ref = reference category (lowest decile).

In addition, survival curves offer a more detailed picture of differing survival rates for different IQ quintiles over the continuous follow-up time (Fig. 3). Namely, participants who scored within the lowest quintile at the age 11 IQ test had under 70% survival probability at age 70, versus 80% survival probability of those who scored in the top IQ quintile. This difference is even more prominent at age 79, where lowest IQ quintile had about 45% chance of survival, versus 65% for those in the top IQ quintile (Fig. 2). This analysis is presented for men and women taken together, and shows a similar graded effect of the IQ as presented in Fig. 3 for men and women separately.

We ran an additional sensitivity analysis. We excluded those participants who scored zero on the MHT (*n* = 619; 377 deceased) because this score could represent an irregularity, rather than a true measure of ability. We ran the baseline model again, with childhood MHT score and age as predictors, and all-cause mortality as an outcome. This did not change the result (HR = 0.80, 95% CI = 0.79, 0.81).

## 4 Discussion

The association between higher childhood intelligence and a reduced risk of all-cause mortality has already been well demonstrated. However, none of the previous studies has been able to examine this association in a near-entire population of both men and women, with a large number of deaths across the whole spectrum of intelligence, and at different ages of death. Our near-entire population sample allowed us to: a) examine whether the result would replicate without a selection bias; b) compare the effect in men and women; c) test whether the association between intelligence and survival is graded across the whole range of IQ; and d) test whether the protective effect of intelligence on mortality differs across different ages of death. Our results suggest that higher childhood intelligence is linked to a higher probability of survival at age 79 in an near-entire year-of-birth cohort. Furthermore, the association between higher IQ and lower mortality risk was overall slightly stronger in women than in men, and in those who died before age 65. Finally, an advantage in each of the IQ quintiles and deciles was associated with an increased hazard ratio for all-cause mortality, i.e. the association between childhood intelligence and mortality across almost 70 years of follow-up is mostly graded.

Our finding that higher childhood IQ is associated with a lower risk of death is in line with those previously reported in a meta-analysis (Calvin et al., 2011). Whereas the overall meta analytic effect size (HR = 0.75) was slightly stronger than ours (HR = 0.80), this may be due to differences in age at intelligence testing and the length of the follow-up period; some studies tested intelligence in young adulthood, and follow-up periods vary. Our effect size is very similar to the meta analytic effect of studies in which IQ was tested between 7 and 12 years (HR = 0.79). Similarly, when only studies with longer follow-up times were included in the meta analysis, the effect size was identical to ours (HR = 0.80) (Calvin et al., 2011).

It is important to understand the reasons for the childhood IQ-mortality association, but such accounts must be based on a robust empirical foundation. At a time of ‘replication crisis’ in psychology, we think that the present report—involving a nearly whole population—provides a secure foundation for the childhood IQ-mortality association. Therefore, there is a solid basis from which to ask why these relationships came about.

### 4.1 Possible mechanisms of the IQ-survival association

A number of models have been proposed to explain (parts of) the association between higher childhood intelligence and reduced mortality risk, as we have enumerated and discussed previously (Deary, 2012, Deary et al., 2010, Whalley and Deary, 2001). For one, higher intelligence has been consistently linked to a variety of health behaviours (Gottfredson & Deary, 2004). For example, higher intelligence has been linked with less cigarette smoking (Batty et al., 2007b, Taylor et al., 2003), less excessive alcohol consumption (Batty, Deary, & Macintyre, 2007), as well as healthier dietary choices and higher levels of physical exercise (Batty, Deary, Schoon, & Gale, 2007a). Some of our current findings could be explained by this mechanism, namely, the differing strength of the association for deaths that occur before and after age 65. One previous study suggested that intelligence may only be associated with early deaths, but not death later in life (Hart et al., 2005), and our results suggest that, whereas the inverse IQ-mortality association may be slightly stronger at younger ages, it is still significant in older age. The slightly stronger association of IQ with earlier deaths might reflect that earlier deaths are more preventable, and possibly associated more strongly with health behaviours that are also associated with intelligence.

Another often discussed mechanism of the IQ-mortality association is the effect of SES, through material advantages rather than health behaviours. With respect to childhood measures, there is good evidence that parental socioeconomic circumstance does not confound the relationship between IQ and mortality (Kilgour, Starr, & Whalley, 2010). However, adjusting for adult SES can attenuate the effects of IQ by up to 30%, suggesting some possible mediation of the IQ-mortality association by SES (Calvin et al., 2011, Kilgour et al., 2010); but it should be noted that childhood IQ also influences adult SES, including education, and social mobility across the life course (Deary et al., 2005).

There could be other factors that influence the association between intelligence and mortality. One possibility is that personality traits act in synergy with intelligence, moderating its association with mortality. Speculatively, this could be an explanation of the small differences between the effect sizes in men and in women observed in the present study. Women on average score higher on conscientiousness (Schmitt, Realo, Voracek, & Allik, 2008), a personality trait strongly implicated in better health outcomes, including mortality (Deary et al., 2010, Jokela et al., 2013, Kern and Friedman, 2008). It is possible that higher IQ enhances beneficial effects of higher concientiousness, forming a particularly good basis for better compliance to health advice and overall increased health behaviours (Bogg and Roberts, 2004, Deary et al., 2010). Since women on average also score higher on the personality trait neuroticism (Schmitt et al., 2008), which has been shown to, in certain cases, have beneficial health effects when interacting with high conscientiousness (Turiano, Mroczek, Moynihan, & Chapman, 2013), it is possible that this configuration of personality traits may further increase the protective effect of intelligence. Furthermore, the interaction between high neuroticism and low intelligence has been shown to be a stronger predictor of mortality than either of the traits alone (Weiss, Gale, Batty, & Deary, 2009). Future studies should further investigate whether different combinations of personality traits interact with IQ in relation to different health behaviours and mortality.

Another possible explanation of the IQ-mortality association that has been put forward is the system-integrity hypothesis (Deary, 2010, Deary, 2012, Whalley and Deary, 2001). This is a notion that higher scores on a cognitive ability test reflect not only more efficient thought processes as a reflection of a biologically more efficient brain, but also body more generally. It is suggested that a ‘better put together body’ exhibits more adaptive reactions to environmental demands, leading to both higher cognitive ability and survival (Deary, 2012). There is substantial evidence that low level physiological measures such as processing speed are a fundamental part of general intelligence (Sheppard & Vernon, 2008), and one that is not influenced by environmental factors such as education (Ritchie, Bates, Der, Starr, & Deary, 2013). Indeed, processing speed (reaction time) accounts for much of the intelligence-mortality association (Deary & Der, 2005), although Deary (2012) suggested that some less cognitive indicator of system integrity would provide a better test of the hypothesis.

In addition to physiological measures as markers of successful functioning of various bodily systems, genetic makeup is a testable candidate that might constitute a foundational aspect of a ‘better put together body’ (Deary, 2012). Similarly to the system integrity hypothesis, Arden, Gottfredson, and Miller (2009) proposed the existence of a “fitness factor”, an index of overall genetic quality that is related to reproductive success and survival. The authors suggested that such a factor could explain part of the association between intelligence and mortality. More recent studies have provided evidence in support of this hypothesis, both from behavioural and molecular genetics angles. For example, a recent study demonstrated that cognitive functions like verbal-numerical reasoning, reaction time, and memory have shared genetic aetiology with adverse health outcomes including BMI, vascular-metabolic diseases and neuropsychiatric diseases (Hagenaars et al., 2016). Such shared genetic aetiology between cognitive ability and risk factors for mortality could explain part of the IQ-mortality associations. Another recent study utilising three large samples of twins estimated that the genetic contribution to the link between intelligence and mortality could be as high as 85–95% (Arden et al., 2015).

These potential explanations are not mutually exclusive, as emphasised in Whalley and Deary (2001). One of the biggest challenges researchers are presented with is to design a study and collect a dataset that would allow for direct tests of these causal models. One step in the right direction is a recent study by Belsky et al. (Belsky et al., 2016), that shows that children with polygenic risk profiles for higher educational attainment were not healthier than those with lower polygenic profiles for education, but had higher cognitive and non-cognitive skills and social mobility. Another GWAS-based study showed that polygenic profiles for higher educational attainment are indeed related to mortality (Marioni et al., 2016). Having genetically informative samples that also contain a breadth of phenotypical data, and a large follow-up time could be key to answering some of the questions posited above. Another possibility is that a quasi-experimental educational reform, such as that described by Brinch and Galloway (Brinch & Galloway, 2012), could be shown to have effects on mortality that are mediated by its IQ-improving effect (see also Lager, Seblova, Falkstedt, & Lövdén, 2016).

### 4.2 Strengths and limitations

The biggest strength of our study is the fact that it has been conducted on a near-entire year-of-birth cohort, therefore containing approximately equal numbers of men and women, a large number of deaths across the whole range of intelligence and ages of death, and almost no selection bias.

The present study also has limitations. The group that is lost to follow-up due to emigration to a foreign country has a higher average IQ than any of the other groups in the sample, likely due to gaining higher qualifications and seeking better employment opportunities elsewhere (Maxwell, 1969). Still, this group comprises a small fraction of our total sample, and does not affect the overall distribution of IQ scores in the sample. Another limitation of the current study is that we were not able to differentiate between causes of death. For example, it is possible that the stronger protective effect of IQ in younger age is driven by earlier deaths being caused by unintentional injury, such as road traffic or industrial accidents (Batty et al., 2009a), or suicide (Batty et al., 2010). This would shed additional light on potential mechanism of the associations. Future studies should investigate associations between intelligence and cause-specific mortality on large samples with long follow-up times. Finally, we note that our data are country and year-of-birth specific, and it cannot be assumed that the results will generalise to other geographical settings and birth-year cohorts, although there are suggestions of similar IQ-mortality associations across these variables too (Calvin et al., 2011).

In conclusion, intelligence is a significant predictor of death in both men and women, especially in younger age. We conclude this from an unusually-definitive study with near-complete tracing of a near-entire year-of-birth population, and with a follow-up period of 68 years.
